# Poster Session I - A103 THE RISK OF VENOUS THROMBOEMBOLISM BY PHASE OF CARE IN PATIENTS WITH INFLAMMATORY BOWEL DISEASE

**DOI:** 10.1093/jcag/gwaf042.103

**Published:** 2026-02-13

**Authors:** J H Chow, A Seeraj, A Corson, G Tennakoon, J McCurdy

**Affiliations:** University of Ottawa Faculty of Medicine, Ottawa, ON, Canada; University of Ottawa Faculty of Medicine, Ottawa, ON, Canada; The Ottawa Hospital Research Institute, Ottawa, ON, Canada; University of Toronto Temerty Faculty of Medicine, Toronto, ON, Canada; University of Ottawa Faculty of Medicine, Ottawa, ON, Canada

## Abstract

**Background:**

Patients with inflammatory bowel disease (IBD) have an increased risk of venous thromboembolism. However, the temporal relationship between VTE and phase of care are poorly understood.

**Aims:**

To determine the proportion of VTE events that are hospital and ambulatory associated.

**Methods:**

We performed a retrospective cohort study at a Canadian tertiary care centre. Adults (≥18 years) with a pre-existing diagnosis of IBD who developed a VTE event between January 1, 2009 and April 31, 2025 were included. Our cohort was identified from our institutional data warehouse using validated administrative codes for IBD and VTE and from our electronic medical records for IBD patients who attended our regional thrombosis clinic. VTE events were categorized as ambulatory or hospital associated. Hospital associated was further categorized as hospital alone, post-discharge alone (within 90 days after discharge), or both. To account for diagnostic delays, VTE events that occurred within 48 hours of hospital admission were considered ambulatory events, and within 48 hours of hospital discharge were considered hospital events.

**Results:**

We identified 393 distinct VTE events involving 338 patients with IBD: 202 (51.4%) males, mean (SD) age of 54.5 (15.9), 232 (59.0%) Crohn’s disease, 155 (39.4%) ulcerative colitis, and 6 (1.5%) IBD-unclassified. The types of VTE events included pulmonary embolism (PE) ± deep vein thrombosis (DVT) (43.8%), DVT alone (32.3%), portal vein thrombosis (10.2%), and other (13.7%). A total of 202 (51.4%) of VTEs were hospital-associated and 191 (48.6%) were ambulatory. The hospital-associated events were further categorized as hospital alone (17.0%), post discharge alone (24.2%), and both (10.2%) (Figure 1). The proportion of patients treated with anticoagulation (prophylaxis or therapeutic) prior to the VTE diagnosis were 8.37% (16/191) ambulatory, 34.3% (23/67) hospital alone, 31.6% (30/95) post discharge alone and 50% (20/40) for both.

**Conclusions:**

Over half of VTE events in patients with IBD were hospital-associated, with the majority occurring during the post-discharge period. This highlights that hospitalization and the post-discharge period are vulnerable times in patients with IBD. Next steps should examine other risk factors to optimize prevention strategies.

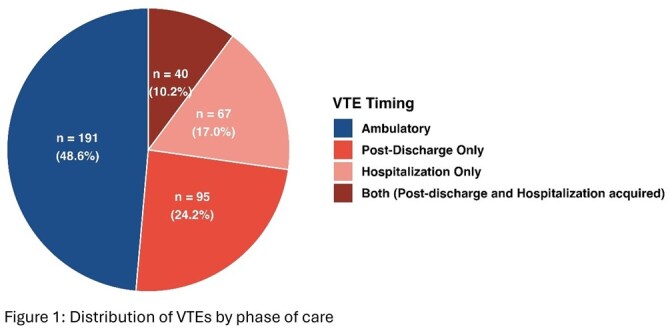

**Funding Agencies:**

None

